# Supplementary material for: Spatiotemporal distribution of power outages with climate events and social vulnerability in the USA
Source: Nat Commun. 2023 Apr 29;14:2470. doi: 10.1038/s41467-023-38084-6 (PMC10147900; doi:10.1038/s41467-023-38084-6)
Supplement: Supplementary file 1 — Supplementary Information [file 41467_2023_38084_MOESM1_ESM.pdf]

Supplementary Information: Spatiotemporal distribution of power outages with climate events and social vulnerability in the USA

Supplementary Methods 1. Steps to generate reliability metrics. POUS, PowerOutage.us, from who we purchased power outage data.

1.1 Estimate population served in each county

- Obtain EIA data (Customers excel documents) of appropriate year here: https://www.eia.gov/electricity/data/eia861/
- Exclude reports of customers related to Part B – "Energy-Only Services". Customers reported for Part B are the same as those reported for Part C – "TDU". They are both part of the same power delivery pipeline (distribution and delivery), and we only kept Part C.
- Flag the customer numbers related to Part D – "RPM + TDU". Texas has a different system and reports customers for retail marketers and transmission distributions jointly rather than other states that report Part A – "RPM" and Part C – "TDU". To retain customer data for Texas, we flag Part D.
- Calculate the total customers per state per utility per year as Part A + Part C for non-Texas states and Part D for Texas.
- Keep customer values only if the combination of state-utility-year appears in the POUS dataset. If a combination does not appear in POUS, then including them in our customer counts will overestimate our values for total customers served by our POUS utilities.
- Obtain county level data about the number of households from the 2015-2019 ACS.
- Obtain county level data about establishments about the county business patterns from the Census Bureau, which was released in 2021.
- Merge county level data about household numbers and establishment numbers by fips.
- Calculate estimates for the total customers served at the state level by summing values about households and establishments. We assume that households and establishments approximate electricity customers.
- Generate proportions for each county within the state as (total households and establishments in a county)/(total households and establishments in the state). These proportions represent the percentage of customers out in a county relative to all customers out in the state.
- Scale down customers from the state to the county level by multiplying the proportions generated in step 10 and the total customers reported for that state.

1.2 Determine reliable API reporting for a county

- Background: Because API data are reported at the utility-subcounty level (e.g., utility A in city A, utility B in city A, utility C in city A), we want to identify APIs that are reliably reporting information. An extreme case that does occur is that an API at a subcounty level reports outage data only once during the entire 3-year study period from 2017 – 2020. We would want to exclude this information from our analysis as data from such APIs are not accurately reporting customers without power.

- General steps:
  - For each utility-subcounty, identify the first and last date time, which will be the utility-subcounty report time.
  - Check the frequency with which the utility-subcounty API reports. The frequency is the total number of observations between the first and last date time.
  - If the frequency (total observations) of a utility-subcounty is less than or equal to 50% of the utility-subcounty report time, then we consider that utility-subcounty API unreliable and exclude.
  - For the remaining utility-subcounty combinations, aggregate metrics (e.g., customers without power) to the county hour.
- Notes:
  - When an API is nonresponsive, which according to POUS documentation is indicated by a duplicated subcounty-utility-date, then we assume that the last observation is carried forward for a maximum of 4 hours.
  - When an API reports a high customer out number for a suspiciously long time (e.g., 90% customers without power for 60 days), we force the value to return to baseline (median) customers without power for that subcounty-utility.

1.3 Determine reliable customer coverage for a county

- Background: Although POUS data has information about customers served, the variable is not reliable on its own. We create a metric as a proxy for how many customers are covered in our POUS data at the county level.
- General steps:
  - For each county, extract the maximum number of customers served reported from the POUS dataset and maximum number of customers downscaled from the state to the county (generated from “Estimate population served in each county”)
  - Calculate the proportion covered as the maximum customers served total (numerator) over the maximum downscaled customer estimates (denominator).
  - Apply a threshold for the proportion of customers covered. For our study, we used a 50% minimum threshold.

Supplementary Methods 2. Description of severe weather and climate event data sources, temporal scale, study definition, and comments/concerns/caveats.

1. Tropical Cyclones
- Data source: IBTrACS
- Temporal scale: 3-hour resolution (aggregated to daily)
- Current definition: Counties are assigned as "exposed" if they cross a 100 km buffer of a given cyclone track at any point in a day
- Comments/concerns/caveats: The 100 km buffer is imperfect as hurricane exposure comprises several heterogeneous exposures (e.g., wind, tornados, precipitation, etc.) Literature used to decide on this buffer includes Anderson et al. and Grabich et al.

2. Lightning
- Data source: International Space Station (ISS) Lightning Imaging Sensor (LIS)
- Temporal scale: time of occurrence
- Current definition: Counties are considered exposed if a lightning flash occurs within the county boundaries at any point of the day. Timezone originally in UTC.
- Comments/concerns/caveats: None.

3. Snowfall
- Data source: National Gridded Snowfall Estimates
- Temporal scale: daily (noon to noon)
- Current definition: Snowfall is defined as days in which snow accumulation aggregated to counties via spatial weighting was equal to or greater than 1 inch.
- Comments/concerns/caveats: None.

4. Anomalous Heat/Cold
- Data source: PRISM
- Temporal scale: Daily (1200-1200/noon-noon UTC)
- Current definition: Anomalous heat exposure is defined as a day above 24 C and that is above the 85th percentile based on average weekly temperature over 30 years (1981-2010). Anomalous cold defined as when temperatures reached below 0° C and below the 15th percentile based on average weekly temperature over the same 30 year period (1981-2010).
- Comments/concerns/caveats: None.

5. Wildfires
- Data source: NIFC
- Temporal scale: Daily
- Current definition: Wildfire exposure is defined as a day in which a county intersects a $\geq$1 km^2 wildfire from the date of discovery to the containment.

- Comments/concerns/caveats: Controlled burns are excluded from the exposure data set at this time. Temporal data for this dataset is limited, and it has been supplemented by data from NIFC. In cases in which a discovery date but no containment data is available, fire out date or control date are used. If no end date is available, multivariate imputation by chained equations was used (using imputation by classification and regression trees method). Furthermore, 159 records that were not successfully linked to the discovery date or containment date were manually searched using the NFIC, CAL FIRE FRAP, and ICS-209-PLUS. These boundaries are static and do not capture the dynamism of the fire boundaries.

6. Heavy Precipitation

- Data source: PRISM
- Temporal scale: Daily (1200-1200/noon-noon UTC)
- Current definition: Daily total, gridded precipitation totals were aggregated to counties via spatial weighting. Heavy precipitation defined as > 85th percentile for a specific county.
- Comments/concerns/caveats: None.

7. Multiple Events

- Data source: NA
- Temporal scale: daily
- Current definition: Multiple events are defined as county days with greater than one severe weather event, that is, a county that experiences more than one of the following in a day: a tropical cyclone, lightning strike, snowfall, anomalous heat or cold, a wildfire, or heavy precipitation.
- Comments/concerns/caveats: None.

Supplementary Figure 1. County threshold of customers out to determine power outage event. Exceeding threshold of customers out meant a county experienced an outage. The threshold based on 0.1% customers out, corresponding to the 90th percentile of customers out per hour at the county-level during the study period. Transparent counties (NA) indicate that 8+ hour outages did not occur there. Power outage data was purchased from PowerOutage.us and county basemaps were obtained from the usmap R package version 0.6.1.

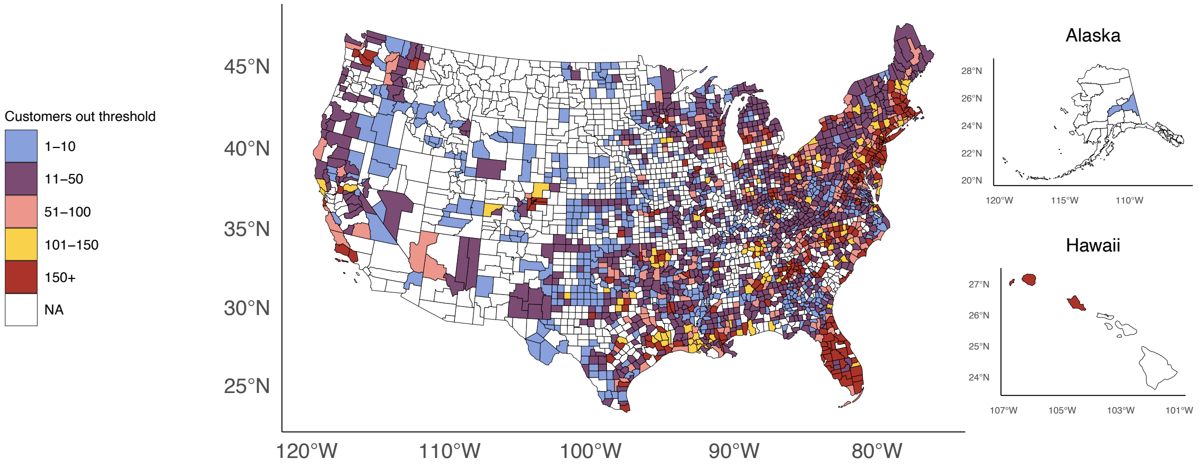

Supplementary Figure 2. Contour plot of the regional counts of 8+ hour outages according to start time of outage per month. Transparent areas indicate that 8+ hour outages did not occur in that region at that time. Power outage data was purchased from PowerOutage.us.

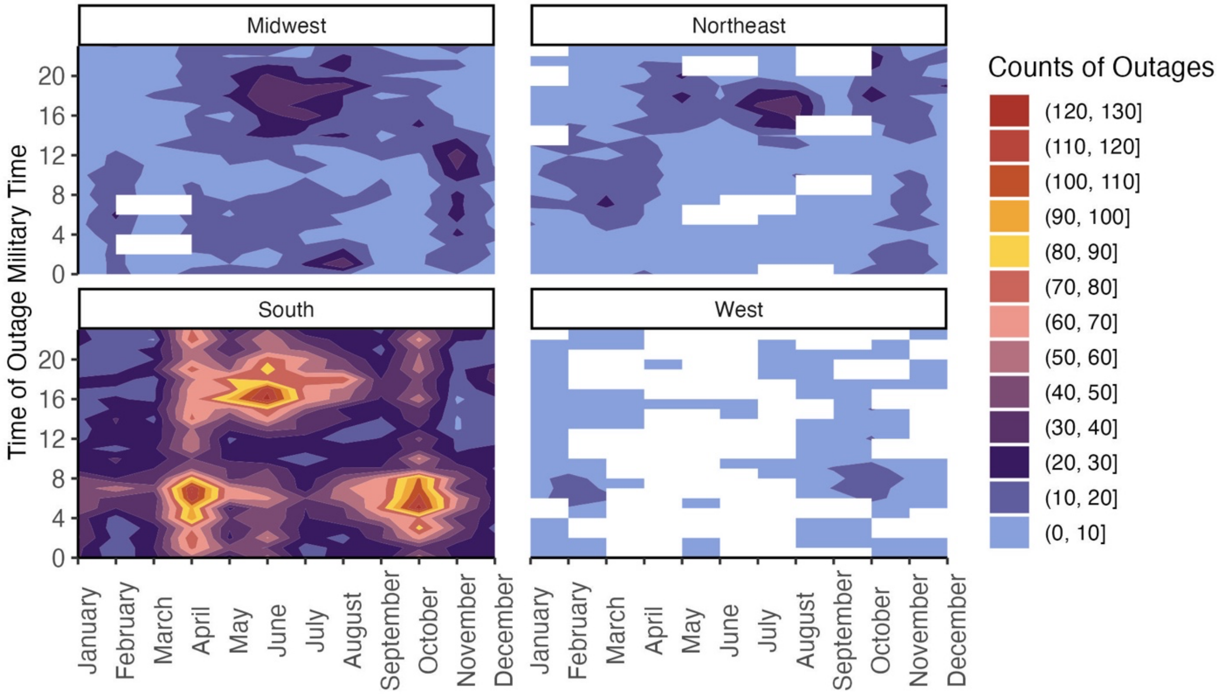

Supplementary Figure 3. Total monthly counts of 8+ hour outages across the study period for counties with 2+ years of data. States with the highest total counts of 8+ hour outages were highlighted. Power outage data was purchased from PowerOutage.us.

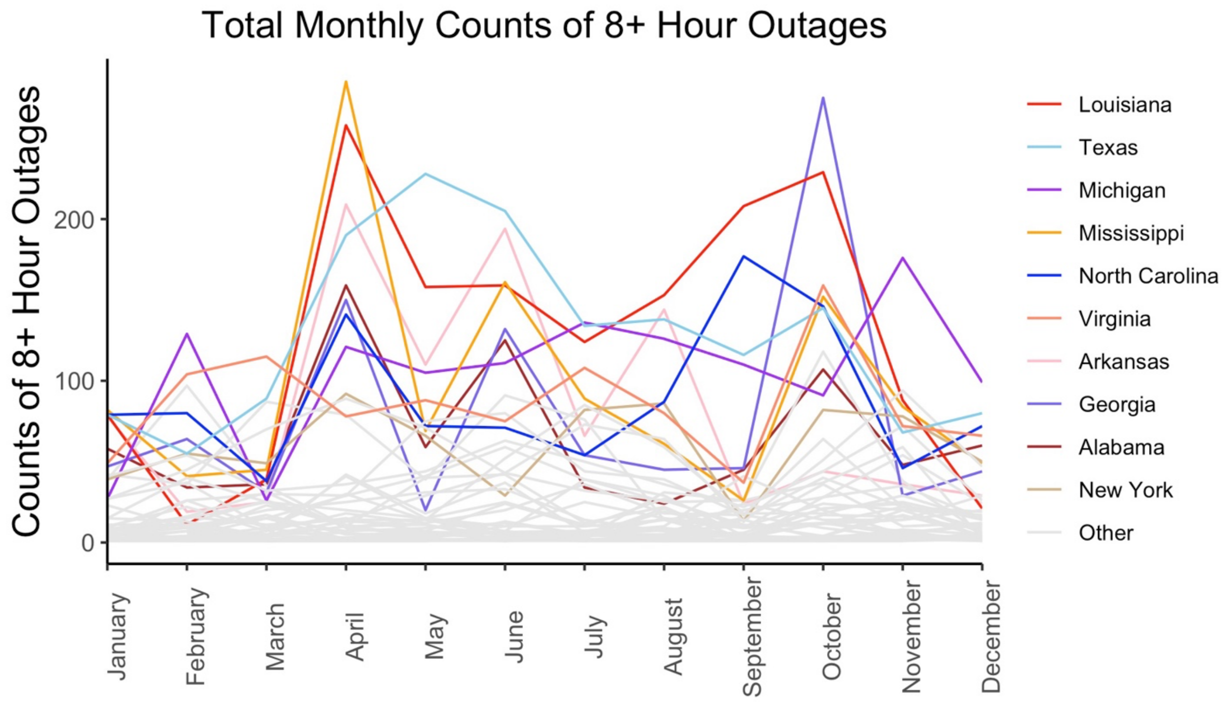

Supplementary Figure 4. Census region monthly distribution of severe weather or climate events on days they co-occurred with 8+ hour outages among counties with 3 years of data. **a** Isolated severe weather and climate events (n = 11,310 county-days). **b** Multiple severe weather and climate event combinations (n = 2,846 county-days). An isolated event was a county-day where only a single severe weather or climate event occurred, and multiple events were county-days where more than one event occurred. We defined a county exposed to lightning if a lightning flash happened, tropical cyclone if the county was within 100km of a cyclone path, wildfire if the county intersects a ≥1 km^2 wildfire, anomalous heat if temperatures exceed 24°C and that is above the 85th percentile, anomalous cold if temperatures are below 0° C and below the 15th percentile, snowfall if snow accumulation > 1 inch, and heavy precipitation if precipitation > 85th percentile. Data used to generate plots are described in Supplementary Methods 2. Transparent areas indicate no weather events co-occurred with outages in that region during that time. Power outage data was purchased from PowerOutage.us.

a

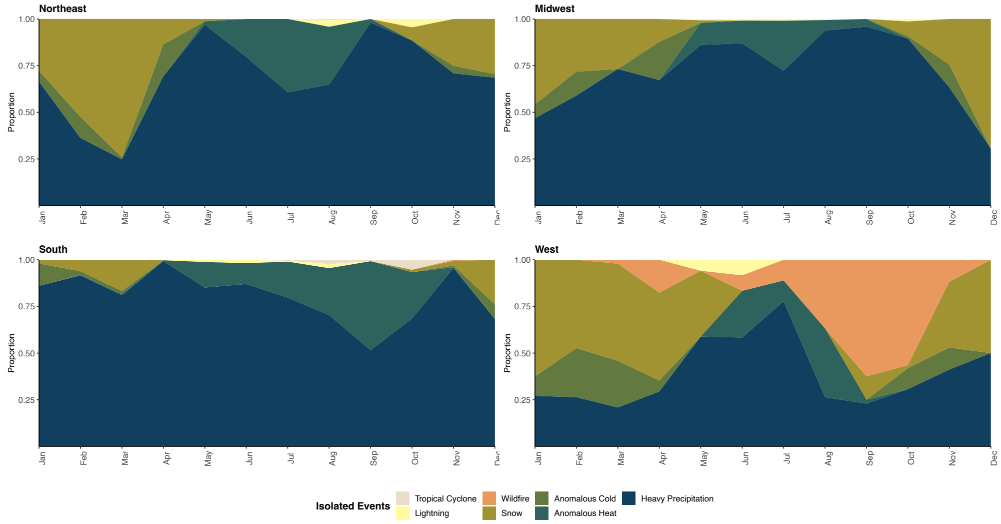

b

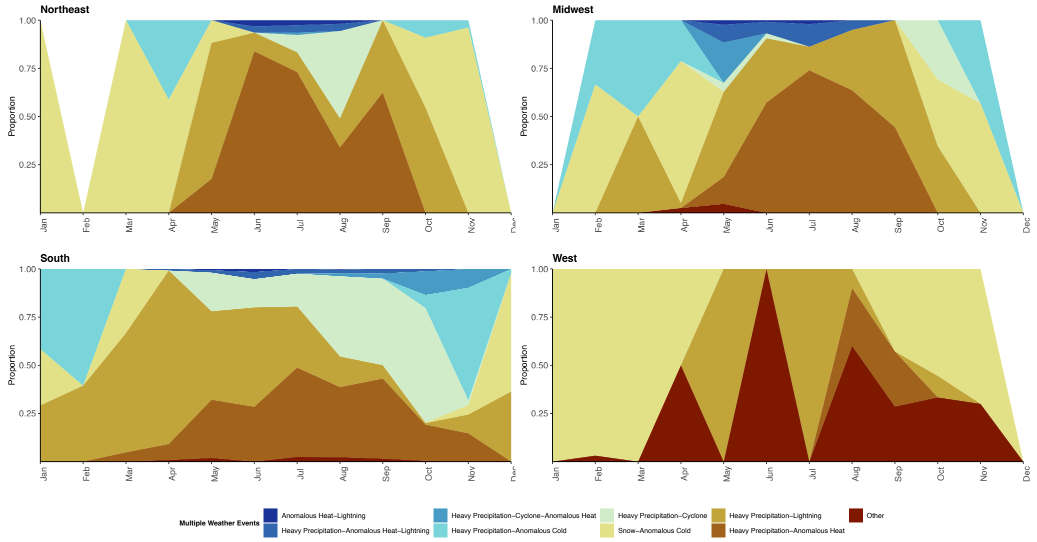

Supplementary Figure 5. Counties classified by their vulnerability metric among counties with 2+ years of reliable data (n = 2,038). a SVI (social vulnerability index) quartile shows that counties with the highest SVI occur in parts of the West coast but largely in the Southeast and South. SVI data are publicly available at https://www.atsdr.cdc.gov/placeandhealth/svi/data_documentation_download.html. **b** Medicare DME (durable medical equipment) use prevalence per 1000 Medicare beneficiaries by quartile illustrates that counties with the highest prevalence exist in the Mountain West and Appalachia. Medicare DME use data are publicly available at https://empowerprogram.hhs.gov/empowermap. Power outage data was purchased from PowerOutage.us and county basemaps were obtained from the usmap R package version 0.6.1.

a

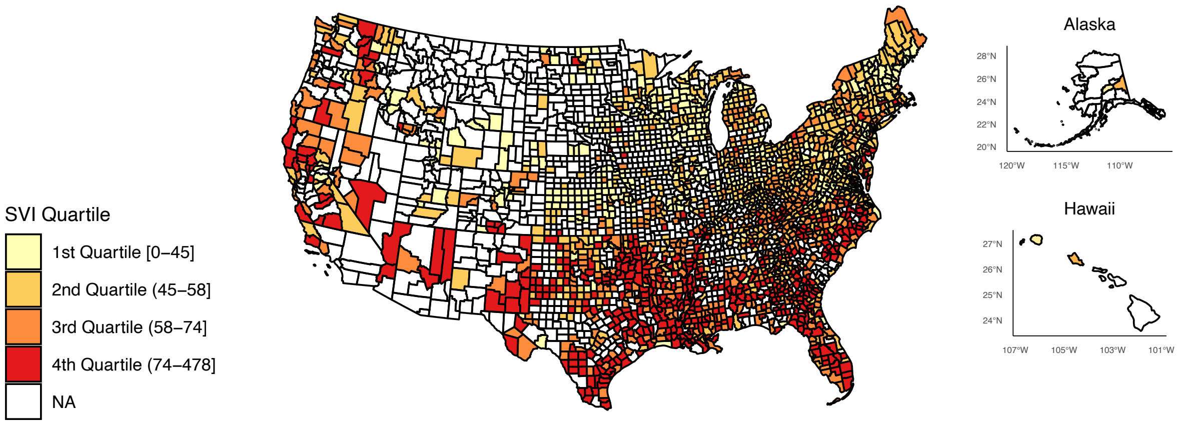

b

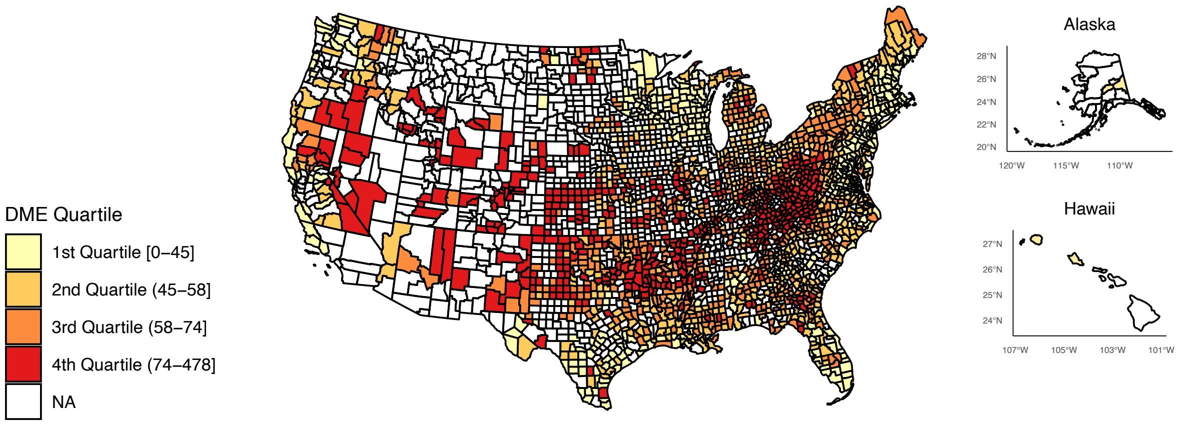

Supplementary Figure 6. Contour plot showing the temporal distribution of 8+ hour outage start times by county SVI quartile and US census region. Transparent areas indicate that 8+ hour outages did not occur in that region at that time. Power outage data was purchased from PowerOutage.us.

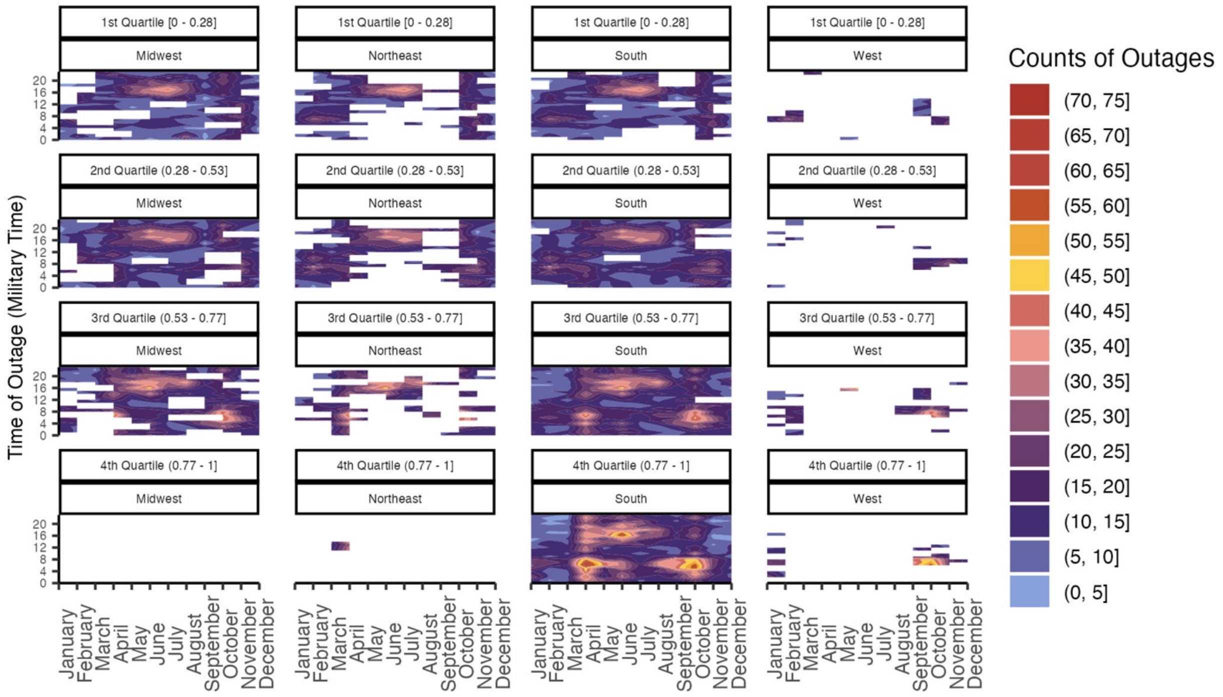

Supplementary Table 1. Description of total counties in the US and counts of total yearly availability of reliable power outage data. Power outage data was purchased from PowerOutage.us.

State	Total Counties	Counties with 3 years of reliable data	Counties with 2 years of reliable data	Counties with 1 years of reliable data	Counties with 0 years of reliable data
AL	67	44 (66%)	5 (7%)	4 (6%)	14 (21%)
AK	29	2 (7%)	0 (%)	0 (%)	27 (93%)
AZ	15	5 (33%)	1 (7%)	0 (%)	9 (60%)
AR	75	54 (72%)	10 (13%)	7 (9%)	4 (5%)
CA	58	22 (38%)	16 (28%)	2 (3%)	18 (31%)
CO	64	12 (19%)	10 (16%)	14 (22%)	28 (44%)
CT	8	8 (100%)	0 (%)	0 (%)	0 (%)
DE	3	3 (100%)	0 (%)	0 (%)	0 (%)
DC	1	1 (100%)	0 (%)	0 (%)	0 (%)
FL	67	41 (61%)	14 (21%)	9 (13%)	3 (4%)
GA	159	107 (67%)	23 (14%)	10 (6%)	19 (12%)
HI	5	2 (40%)	0 (%)	0 (%)	3 (60%)
ID	44	10 (23%)	7 (16%)	14 (32%)	13 (30%)
IL	102	43 (42%)	7 (7%)	32 (31%)	20 (20%)
IN	92	51 (55%)	19 (21%)	16 (17%)	6 (7%)
IA	99	14 (14%)	17 (17%)	43 (43%)	25 (25%)
KS	105	34 (32%)	28 (27%)	12 (11%)	31 (30%)
KY	120	79 (66%)	8 (7%)	20 (17%)	13 (11%)
LA	64	51 (80%)	9 (14%)	0 (%)	4 (6%)
ME	16	16 (100%)	0 (%)	0 (%)	0 (%)
MD	24	24 (100%)	0 (%)	0 (%)	0 (%)
MA	14	10 (71%)	4 (29%)	0 (%)	0 (%)
MI	83	51 (61%)	17 (20%)	2 (2%)	13 (16%)
MN	87	30 (34%)	13 (15%)	8 (9%)	36 (41%)
MS	82	53 (65%)	1 (1%)	7 (9%)	21 (26%)
MO	115	27 (23%)	22 (19%)	52 (45%)	14 (12%)
MT	56	0 (%)	0 (%)	1 (2%)	55 (98%)
NE	93	13 (14%)	15 (16%)	5 (5%)	60 (65%)
NV	17	4 (24%)	2 (12%)	1 (6%)	10 (59%)
NH	10	10 (100%)	0 (%)	0 (%)	0 (%)
NJ	21	21 (100%)	0 (%)	0 (%)	0 (%)
NM	33	8 (24%)	3 (9%)	3 (9%)	19 (58%)
NY	62	62 (100%)	0 (%)	0 (%)	0 (%)

NC	100	75 (75%)	11 (11%)	9 (9%)	5 (5%)
ND	53	3 (6%)	13 (25%)	6 (11%)	31 (58%)
OH	88	72 (82%)	9 (10%)	4 (5%)	3 (3%)
OK	77	48 (62%)	18 (23%)	5 (6%)	6 (8%)
OR	36	15 (42%)	3 (8%)	8 (22%)	10 (28%)
PA	67	64 (96%)	1 (1%)	1 (1%)	1 (1%)
RI	5	5 (100%)	0 (%)	0 (%)	0 (%)
SC	46	16 (35%)	6 (13%)	23 (50%)	1 (2%)
SD	66	2 (3%)	8 (12%)	14 (21%)	42 (64%)
TN	95	31 (33%)	21 (22%)	13 (14%)	30 (32%)
TX	254	142 (56%)	19 (7%)	46 (18%)	47 (19%)
UT	29	5 (17%)	2 (7%)	1 (3%)	21 (72%)
VT	14	10 (71%)	3 (21%)	0 (%)	1 (7%)
VA	133	120 (90%)	4 (3%)	2 (2%)	7 (5%)
WA	39	15 (38%)	9 (23%)	5 (13%)	10 (26%)
WV	55	55 (100%)	0 (%)	0 (%)	0 (%)
WI	72	61 (85%)	1 (1%)	9 (13%)	1 (1%)
WY	23	6 (26%)	2 (9%)	1 (4%)	14 (61%)

Supplementary Table 2. Top 5 states by total outage count, counties with outage counts in top decile, and percentage of counties in top decile. Power outage data was purchased from PowerOutage.us.

Total Outage Count		States with the greatest number of counties with Outage Counts in Top Decile	
8+ Hour	1+ Hour	8+ Hour	1+ Hour
Louisiana (553)	Texas (11,504)	Michigan (32)	Texas (37)
Texas (527)	Georgia (10,609)	Louisiana (29)	Louisiana (28)
Michigan (447)	Louisiana (7,826)	Mississippi (18)	Alabama (22)
Mississippi (381)	Mississippi (7,188)	Texas (18)	Mississippi (22)
North Carolina (372)	Alabama (6,240)	New York and Virginia (15)	Pennsylvania and Georgia (14)

Supplementary Table 3. Top 5 States with highest total customer-time without power and top 5 counties with highest total customer-time without power during our study period. Power outage data was purchased from PowerOutage.us.

Geography	Total Average Customer-Time Without Power
State	
Louisiana	52,182,771
North Carolina	38,267,104
California	30,281,140
Texas	30,027,869
New York	28,780,714
County	
Calcasieu (LA)	10,045,051
Los Angeles (CA)	7,815,756
Fairfield (CT)	7,551,849
Davidson (TN)	7,361,656
Jefferson (LA)	6,156,641

Supplementary Table 4. County-day co-occurrence of severe weather or climate events and 8+ hour outages, including all combinations of multiple events. Analysis included the 1,653 continental US counties with 3 years of reliable data. We defined co-occurrence if the weather or climate event occurred in the same county on the same day the 8+ hour outage began. Note that we separated snowfall out from heavy precipitation. [a] The co-occurrence ratio was computed as the proportion of county-days with severe weather or climate event type *i* that co-occurred with an 8+ hour outage divided by the proportion of county-days without any weather or climate event that co-occurred with an 8+ hour outage. A co-occurrence ratio > 1 means that 8+ hour outages were more likely to occur on county-days with severe weather or climate event *i* compared to days with no event and a ratio < 1 means that 8+ hour outages were less likely to occur on county-days with severe weather or climate event *i* compared to days with no event. [b] An isolated event was a county-day where only a single severe weather or climate event occurred. We defined a county exposed to lightning if a lightning flash happened, tropical cyclone if the county was within 100km of a cyclone path, wildfire if the county intersects a ≥1 km^2 wildfire, anomalous heat if temperatures exceed 24°C and that is above the 85th percentile, anomalous cold if temperatures are below 0° C and below the 15th percentile, snowfall if snow accumulation > 1 inch, and heavy precipitation if precipitation > 85th percentile. [c] Multiple events indicate county-days where multiple severe weather or climate events occurred. Power outage data was purchased from PowerOutage.us.

Severe Weather or Climate Event	County-Days, N (%)	8+ Hour Outage County-Days, N (%)	Co-occurrence Ratio [a]
Total	1,799,319 (100.0)	22,793 (100.0)	-
None	1,265,181 (70.3)	8,629 (37.9)	0.5
None	1,265,213 (70.2)	8,637 (37.9)	-
Isolated event[b]	492,489 (27.4)	11,310 (49.7)	3.4
Heavy precipitation	267,823 (14.9)	8,507 (37.3)	4.7
Snowfall	25,523 (1.4)	1,172 (5.1)	6.7
Anomalous heat	131,727 (7.3)	1,090 (4.8)	1.2
Anomalous cold	57,924 (3.2)	344 (1.5)	0.9
Wildfire	5,381 (0.3)	84 (0.4)	2.3
Lightning	3,578 (0.2)	63 (0.3)	2.6
Multiple Events[c]	41,576 (2.3)	2,846 (12.5)	5.4
Heavy Precipitation-Anomalous Heat	17,415 (1.0)	917 (4.0)	7.7
Heavy Precipitation-Cyclone	2,650 (0.2)	679 (3.0)	37.6
Heavy Precipitation-	8,142 (0.5)	644 (2.8)	11.6

Lightning			
Snowfall-<br>Anomalous Cold	7,151 (0.4)	250 (1.1)	5.1
Heavy Precipitation-<br>Anomalous Cold	1,779 (0.1)	134 (0.6)	11.0
Heavy Precipitation-<br>Cyclone-<br>Anomalous Heat	244 (0.0)	86 (0.4)	51.7
Heavy Precipitation-<br>Anomalous Heat-<br>Lightning	1,197 (0.1)	78 (0.3)	9.5
Anomalous Heat-<br>Lightning	985 (0.1)	16 (0.1)	2.4
Heavy Precipitation-<br>Cyclone-<br>Lightning	39 (0.0)	14 (0.1)	52.6
Heavy Precipitation-<br>Wildfire	949 (0.1)	9 (0.0)	1.4
Anomalous Heat-<br>Wildfire	570 (0.0)	6 (0.0)	1.5
Snowfall-<br>Lightning	24 (0.0)	4 (0.0)	24.4
Snowfall -<br>Wildfire	58 (0.0)	3 (0.0)	7.6
Cyclone-<br>Anomalous Heat	41 (0.0)	3 (0.0)	10.7
Snowfall -<br>Anomalous Cold-<br>Wildfire	7 (0.0)	0 (0.0)	0.0
Anomalous Cold-<br>Wildfire	60 (0.0)	1 (0.0)	2.4
Heavy Precipitation-<br>Cyclone-<br>Anomalous Heat-<br>Lightning	5 (0.0)	1 (0.0)	29.3
Heavy Precipitation-<br>Anomalous Heat-<br>Wildfire	141 (0.0)	0 (0.0)	0.0
Lightning-<br>Wildfire	72 (0.0)	0 (0.0)	0.0
Heavy Precipitation-	48 (0.0)	0 (0.0)	0.0

Lightning-<br>Wildfire			
Anomalous Heat-<br>Lightning-<br>Wildfire	25 (0.0)	0 (0.0)	0.0
Anomalous Cold-<br>Snowfall -<br>Lightning	7 (0.0)	0 (0.0)	0.0
Anomalous Cold-<br>Lightning	2 (0.0)	0 (0.0)	0.0
Heavy Precipitation-<br>Anomalous Cold-<br>Lightning	2 (0.0)	0 (0.0)	0.0
Heavy Precipitation-<br>Anomalous Heat-<br>Lightning-<br>Wildfire	2 (0.0)	1 (0.0)	73.3
Cyclone-<br>Anomalous Heat-<br>Wildfire	1 (0.0)	0 (0.0)	0.0
Heavy Precipitation-<br>Anomalous Cold-<br>Wildfire	1 (0.0)	0 (0.0)	0.0

Supplementary Table 5. Attributes of counties with high SVI and high 8+ hour outage exposure. *denotes p-value < 0.05 from two-sided t-test comparison of SVI, social vulnerability index from the Centers for Disease Control and Prevention and Agency for Toxic Substances and Disease Registry. Power outage data was purchased from PowerOutage.us.

SVI Theme	Variable	High-High Cluster	All Other Clusters	Difference	P-value
Socioeconomic Status	Below 150% Poverty (%)*	34.0	24.3	9.7	<0.01
	Unemployed (%)*	7.1	5.2	2.0	<0.01
	Housing Cost Burden (%)	22.7	22.3	0.5	0.5
	No High School Diploma (%)*	16.2	12.3	3.9	<0.01
	No Health Insurance (%)	9.8	9.5	0.3	0.5
Household Characteristics	Aged 65 and Older (%)*	17.9	19.3	-1.3	<0.01
Racial/Ethnic Minority Status	Aged 17 and Younger (%)	22.5	22.1	0.4	0.2
	Civilian with a Disability (%)*	18.2	16.0	2.2	<0.01
	Single-Parent Household (%)*	8.0	5.8	2.2	<0.01
	English Language Proficiency (%)*	1.0	1.6	-0.7	<0.01
	Racial and Ethnic Minority (%)*	40.4	23.9	16.5	<0.01
Housing Type & Transportation	Multi-Unit Homes (%)*	3.3	4.8	-1.5	<0.01
	Mobile Homes (%)*	20.1	12.4	7.7	<0.01
	Crowding (%)	2.4	2.4	0.0	0.9
	No Vehicle (%)*	8.1	6.1	2.0	<0.01
	Group Quarters (%)*	5.4	3.5	2.0	<0.01
