# Supplementary material for: Spatiotemporal distribution of power outages with climate events and social vulnerability in the USA
Source: Nat Commun. 2023 Apr 29;14:2470. doi: 10.1038/s41467-023-38084-6 (PMC10147900; doi:10.1038/s41467-023-38084-6)
Supplement: Supplementary file 3 — Reporting Summary [file 41467_2023_38084_MOESM3_ESM.pdf]

nature portfolio

Corresponding author(s): Joan A. Casey

Last updated by author(s): March 28, 2023

Reporting Summary

Nature Portfolio wishes to improve the reproducibility of the work that we publish. This form provides structure for consistency and transparency in reporting. For further information on Nature Portfolio policies, see our Editorial Policies and the Editorial Policy Checklist.

Please do not complete any field with "not applicable" or n/a. Refer to the help text for what text to use if an item is not relevant to your study.
For final submission: please carefully check your responses for accuracy; you will not be able to make changes later.

Statistics

For all statistical analyses, confirm that the following items are present in the figure legend, table legend, main text, or Methods section.

n/a	Confirmed	
☐	☒	The exact sample size (*n*) for each experimental group/condition, given as a discrete number and unit of measurement
☒	☐	A statement on whether measurements were taken from distinct samples or whether the same sample was measured repeatedly
☐	☒	The statistical test(s) used AND whether they are one- or two-sided<br>*Only common tests should be described solely by name; describe more complex techniques in the Methods section.*
☐	☒	A description of all covariates tested
☐	☒	A description of any assumptions or corrections, such as tests of normality and adjustment for multiple comparisons
☐	☒	A full description of the statistical parameters including central tendency (e.g. means) or other basic estimates (e.g. regression coefficient) AND variation (e.g. standard deviation) or associated estimates of uncertainty (e.g. confidence intervals)
☐	☒	For null hypothesis testing, the test statistic (e.g. *F*, *t*, *r*) with confidence intervals, effect sizes, degrees of freedom and *P* value noted<br>*Give P values as exact values whenever suitable.*
☒	☐	For Bayesian analysis, information on the choice of priors and Markov chain Monte Carlo settings
☒	☐	For hierarchical and complex designs, identification of the appropriate level for tests and full reporting of outcomes
☒	☐	Estimates of effect sizes (e.g. Cohen's *d*, Pearson's *r*), indicating how they were calculated

Our web collection on statistics for biologists contains articles on many of the points above.

Software and code

Policy information about availability of computer code

Data collection	No software was used to collect these data.
Data analysis	We used R Version 4.1.0 (2021-05-18) for all analyses. For bivariate local indicators for spatial analysis, we used the rgeoda package version 0.0.9 mentioned in the manuscript with further links in the reference section. Analytic code is available at https://github.c om/viviando/ National-Power-Outages. For our national maps, we used the usmap R package version 0.6.1, which is mentioned in the manuscript.

For manuscripts utilizing custom algorithms or software that are central to the research but not yet described in published literature, software must be made available to editors and reviewers. We strongly encourage code deposition in a community repository (e.g. GitHub). See the Nature Portfolio guidelines for submitting code & software for further information.

Data

Policy information about availability of data

All manuscripts must include a data availability statement. This statement should provide the following information, where applicable:

- Accession codes, unique identifiers, or web links for publicly available datasets
- A description of any restrictions on data availability
- For clinical datasets or third party data, please ensure that the statement adheres to our policy

The power outage data that support the findings of this study are available for purchase from PowerOutage.US at https://power outage.us/products. Processed data containing annual average counts of outage events and customers without power are available at https://github.com/viviando/National-Power-Outages. The

Centers for Disease Control and Prevention Social Vulnerability Index data is available publicly at https://www.atsdr.cdc.gov/placeandhealth/svi/data_documentation_download.html and the Health and Human Services Medicare durable medical equipment data is available for download at https://empowerprogram.hhs.gov/empowermap. A full description of data used to generate severe weather and climate events is available in Supplementary Information Methods 2.

Human research participants

Policy information about studies involving human research participants and Sex and Gender in Research.

Reporting on sex and gender

Our study does not report on sex and gender as we report customers without power at the county level.

Population characteristics

Social Vulnerability Index: we used the Centers for Disease Control and Prevention's 2016-2020 Social Vulnerability Index, which is generated at the county-level based on 16 U.S. census variables and is publicly-available for download.

Medicare durable medical equipment users: We used the Health and Human Services summary of the count of Medicare beneficiaries using durable medical equipment and overall number of Medicare beneficiaries per county. Medicare beneficiaries in those that were currently enrolled in the Centers for Medicare and Medicaid Service Medicare Fee-For-Service (Parts A/B) or Medicare Advantage (Part C). These data are publicly-available for download.

Both datasets were available for study counties with 2+ years of reliable outage data (n=2038).

Recruitment

These are secondary data and thus no recruitment took place.

Ethics oversight

The Columbia University Institutional Review Board approved this research (Protocol #AAAT5765).

Note that full information on the approval of the study protocol must also be provided in the manuscript.

Field-specific reporting

Please select the one below that is the best fit for your research. If you are not sure, read the appropriate sections before making your selection.

☒ Life sciences ☐ Behavioural & social sciences ☐ Ecological, evolutionary & environmental sciences

For a reference copy of the document with all sections, see nature.com/documents/nr-reporting-summary-flat.pdf

Life sciences study design

All studies must disclose on these points even when the disclosure is negative.

Sample size

The study used a power outage dataset that covered the entire United States and was purchased from PowerOutage.US. PowerOutage.US gathers outage data at subcounty levels such as cities or towns using utility providers' application programming interfaces. No sample size calculations were performed. As we aimed to aggregate this data to the county and hourly level, we evaluated temporal and spatial missing-ness and, instead, applied several thresholds to ensure data reliability. Namely, we considered a county's data to be unreliable and excluded the county from our study if (1) the API response was less than or equal to 50% of the report time for that county and/or (2) the customer coverage was less than 50% of the total county customers. Criteria (1) and (2) were determined a priori. The final sample contained a total of 2,447 U.S. counties from 2018-2020 with 1, 2, or 3 years of reliable data. There were 2,447 counties with 1+ years of data, 2,038 counties with 2+ years of data, and 1,653 counties with 3+ years of data. Data years available for every county in our dataset is discussed throughout the manuscript. Further details about the process can be found in the Supplement and in the GitHub repository.

For meteorologic datasets (i.e. - International Best Track Archive for Climate Stewardship (IBTrACS), International Space Station (ISS) Lightning Imaging Sensor (LIS), National Gridded Snowfall Estimates, PRISM, National Interagency Fire Center), we extracted data for every day in our study period, 2018-2020. We linked this data to counties with 3 years of data (N = 2,447 counties).

For the social vulnerability and medical vulnerability data, we included such information (i.e. – SVI scores, durable medical equipment use prevalence) for counties in our dataset that had 2+ years of reliable data (N = 2,038 counties).

Data exclusions

Data from two utilities (serving 31 counties) were confirmed incomplete by PowerOutage.US and were excluded from analysis. We applied data quality and reliability checks, retaining reliable counties from certain analyses based on a county utility provider's application programming interface (API) performance. Specifically, we include counties with an API reported ≥ 50% of the time and/or ≥ 50% of total county customers. With these criteria, we excluded 563 (18.7%) counties from the study.

Replication

The final power outage dataset and analyses were successfully replicated by two researchers, undergoing at least two rounds of full review and several rounds of sub-steps for data cleaning.

Data on social and medical vulnerability were not created by this research group and is instead directly downloadable on public forums.

Meteorological data were collected and aggregated by two researchers who replicated and reviewed the finalized dataset successfully.

Randomization

Not relevant because this was an observational study.

Blinding	Not relevant because this was an observational study.

Reporting for specific materials, systems and methods

We require information from authors about some types of materials, experimental systems and methods used in many studies. Here, indicate whether each material, system or method listed is relevant to your study. If you are not sure if a list item applies to your research, read the appropriate section before selecting a response.

Materials & experimental systems

n/a	Involved in the study
☒	☐ Antibodies
☒	☐ Eukaryotic cell lines
☒	☐ Palaeontology and archaeology
☒	☐ Animals and other organisms
☒	☐ Clinical data
☒	☐ Dual use research of concern

Methods

n/a	Involved in the study
☒	☐ ChIP-seq
☒	☐ Flow cytometry
☒	☐ MRI-based neuroimaging

This checklist template is licensed under a Creative Commons Attribution 4.0 International License, which permits use, sharing, adaptation, distribution and reproduction in any medium or format, as long as you give appropriate credit to the original author(s) and the source, provide a link to the Creative Commons license, and indicate if changes were made. The images or other third party material in this article are included in the article's Creative Commons license, unless indicated otherwise in a credit line to the material. If material is not included in the article's Creative Commons license and your intended use is not permitted by statutory regulation or exceeds the permitted use, you will need to obtain permission directly from the copyright holder. To view a copy of this license, visit http://creativecommons.org/licenses/by/4.0/
